# Metabolic host response and therapeutic approaches to influenza infection

**DOI:** 10.1186/s11658-020-00211-2

**Published:** 2020-03-05

**Authors:** Mohsen Keshavarz, Farid Solaymani-Mohammadi, Haideh Namdari, Yaser Arjeini, Mohammad Javad Mousavi, Farhad Rezaei

**Affiliations:** 1 The Persian Gulf Tropical Medicine Research Center, The Persian Gulf Biomedical Sciences Research Institute, Bushehr University of Medical Sciences, Bushehr, Iran; 2 Department of Biological Sciences, North Dakota State University, Fargo, North Dakota USA; 3 Iranian Tissue Bank and Research Center, Tehran University of Medical Sciences, Tehran, Iran; 4 Department of Virology, School of Public Health, Tehran University of Medical Sciences, Tehran, Iran; 5 Department of Medical Immunology, School of Medicine, Tehran University of Medical Sciences, Tehran, Iran; 6 Department of Immunology and Allergy, Faculty of Medicine, Bushehr University of Medical Sciences, Bushehr, Iran; 7 National Influenza Center, School of Public Health, Tehran University of Medical Sciences, Tehran, Iran

**Keywords:** Influenza, Glycolysis, Fatty acid synthesis, Metabolism, Indoleamine-2,3-dioxygenase

## Abstract

Based on available metabolomic studies, influenza infection affects a variety of cellular metabolic pathways to ensure an optimal environment for its replication and production of viral particles. Following infection, glucose uptake and aerobic glycolysis increase in infected cells continually, which results in higher glucose consumption. The pentose phosphate shunt, as another glucose-consuming pathway, is enhanced by influenza infection to help produce more nucleotides, especially ATP. Regarding lipid species, following infection, levels of triglycerides, phospholipids, and several lipid derivatives undergo perturbations, some of which are associated with inflammatory responses. Also, mitochondrial fatty acid β-oxidation decreases significantly simultaneously with an increase in biosynthesis of fatty acids and membrane lipids. Moreover, essential amino acids are demonstrated to decline in infected tissues due to the production of large amounts of viral and cellular proteins. Immune responses against influenza infection, on the other hand, could significantly affect metabolic pathways. Mainly, interferon (IFN) production following viral infection affects cell function via alteration in amino acid synthesis, membrane composition, and lipid metabolism. Understanding metabolic alterations required for influenza virus replication has revealed novel therapeutic methods based on targeted inhibition of these cellular metabolic pathways.

## Background

Influenza virus infection (IVI) is one of the most common infectious agents, capable of infecting a variety of avian and mammalian species. The virus is responsible for seasonal epidemics, leading to 3–5 million severe infections and 250,000–500,000 deaths annually [1, 2]. Despite the annual vaccination program, the high mortality rate caused by influenza infection and its various complications, including chronic lung disease, cardiac disease, asthma, and metabolic disorders, is yet to be adequately addressed [3–5].

In 1956, Eagle et al. first indicated that the addition of glucose to HeLa cell medium could promote the generation of poliovirus progeny [6]. Results of a published study showed that the replication of the influenza virus depends on host cellular metabolism such that metabolites including nucleic acids, proteins, glycoproteins, and lipids are crucially required for the life cycle of the influenza virus [7]. Recent research on a mouse model showed that influenza infection could affect more than 100 metabolite markers in serum, lung, and bronchoalveolar lavage fluid [8]. Acquiring the required materials from the host cell to self-replicate, the virus can disrupt biochemical processes such as glycolysis, fatty acid (FA) synthesis, and glutamine pathways [9]. It is of particular importance for scientists to broaden their horizon on the metabolic changes during influenza infection, which in turn paves the way for preventing life-threatening consequences.

Influenza infection actively provokes the pro-oxidant condition in the host cell to facilitate viral proliferation and pathogenesis. Increased expression of influenza M2 protein can activate protein kinase C and increase reactive oxygen species (ROS) production [10]. On the other hand, PB1-F2 decreases superoxide anion dismutase 1 (SOD1) expression and consequently disrupts the ROS scavenging process [11]. In people with influenza infection, increased levels of DNA, lipid, and protein oxidation products are found in plasma and urine [12–14]. Also, increased levels of ROS and inducible nitric oxide synthase (iNOS) have been observed as markers of oxidative stress in the lungs of people who died due to pandemic influenza infection [15]. ROS-producing enzymes induced by influenza infection mainly include NADPH oxidase (Nox) and xanthine oxidase, upregulation of which causes the impaired defensive function of antioxidants [16]. An increase in ROS production, along with impaired antioxidant function, ultimately leads to a profound change in redox homeostasis of the cell [16–19]. Nox2 is a phagocytic enzyme that is involved in the production of ROS induced by influenza virus [19–22], and impaired Nox2 expression results in a lack of increased RNS and ROS production following influenza infection [20]. Xanthine oxidase is also an ROS-producing enzyme that is induced by influenza infection [23, 24], and its inhibition can hinder ROS increase in the cell.

On the other hand, increased expression of SOD1 reduces influenza virus titers within the cell [25]. It is also reported that influenza infection significantly increases ROS production by inducing Nox4, and the proliferation of this virus in lung epithelial cells is dependent on redox-sensitive pathways activated by Nox4-derived ROS [16]. Glutathione (GSH) is a vital antioxidant in the cell, and its cellular content is inversely related to influenza virus replication in the cell [26, 27]. It is indicated that higher levels of GSH, antioxidant enzymes such as glutathione peroxidase, and the anti-apoptotic protein Bcl-2 in the lungs of female mice result in superior resistance of these mice to influenza infection. In contrast, male mice are more susceptible to this infection due to higher expression of Nox4. This difference is due primarily to the higher ability of female mice to maintain cellular redox homeostasis [28]. Amatore et al. also showed that an increase in GSH content in organs by affecting GSH-dependent antiviral pathways strengthens the immune system, in particular Th1 cell response, and decreases viral replication [29]. However, GSH depletion results in a deviation of the response towards Th2 cells [30].

Furthermore, oxidative stress following infection can induce the transcription factor NF-kB, which subsequently leads to increased levels of inflammatory cytokines, including interleukin (IL)-1β, IL-6, IFN, and TNF [31, 32]. IFNs are one of these cytokines that trigger and affect T cell metabolism via mediating glucose uptake, glycolysis, and lipid synthesis. IFN can also exert its function on metabolic changes by producing several mediators including indoleamine-2,3-dioxygenase (IDO) and nitric oxide (NO), both of which appear to have either an inducible or an inhibitory role in viral replication [33]. Since tryptophan is critical for T cell proliferation, depletion of this amino acid by IDO suppresses the immune system through the stimulation of T regulatory cells. NO, on the other hand, inhibits viral replication via changes in the structure of viral proteins [34]. In this review, we first discuss the metabolic abnormalities during influenza infection and then shed light on the role of immunometabolites that regulate cellular metabolism. The following sections summarize recent evidence about the novel therapeutic approaches that target metabolic pathways in influenza infection.

### Metabolic perturbations in influenza infection

Viruses take advantage of various cellular mechanisms to replicate efficiently. Vital metabolic pathways of host cells are one of the most widely used mechanisms targeted by viruses, resulting in considerable changes. In this context, studies have revealed that various human viruses, such as cytomegalovirus [35–37], rubella [38, 39], dengue [40], mumps [41], poliovirus [42, 43] and reovirus [44] can strongly affect host cell glycolysis, lipid metabolism, and glutaminolysis. Furthermore, a review by Sanchez and Lagunoff delineated the activation of these metabolic processes by several viruses [45]. As will be discussed in this section, influenza as a highly pathogenic human virus interferes tremendously with the host metabolic cycles and thereby forces them to produce viral particles more efficiently.

### Glucose metabolism

The metabolism and concentration of glucose in the cell play a cardinal role in the homeostasis of cellular metabolic procedures [46]. Shortly after the onset of IVI, the rate of glucose uptake by infected cells increases continually, and the subsequently enhanced glycolysis results in higher glucose consumption, production of viral particles [47, 48], and extracellular concentration of lactate. The relationship between glycolysis and IVI has shown that influenza infection at a higher multiplicity of infection (MOI) raises the glycolytic activity of the cells [49]. In a study by Kohio and Adamson, a dose-specific increase in influenza infection was associated with higher glucose levels, whereas the treatment of cells with glycolysis inhibitors remarkably suppressed the viral replication. However, the viral infection could be retriggered by adding ATP to the cell environment. This study revealed that enhancing vacuolar-type ATPase (a proton pump essential for influenza uncoating) via increasing glucose metabolism and, as a result, higher available ATP resources, augments the virus infection [50]. The observations, as mentioned above, reveal a significant increase in ATP and glucose consumption within cells following influenza infection and also highlight the dependence of the influenza virus on the glycolysis pathway for energy production. The viral replication has the highest use of ATP during influenza infection, releasing large quantities of energy in the form of heat. This process can increase the temperature of infected cells by 4–5 °C. As viral proliferation increases, the cellular ATP level drops sharply, resulting in reduced potential and stability of the mitochondrial membrane [51]. Based on available results, patients with metabolic disorders can develop more severe influenza infection compared to healthy hosts. There have been several studies showing that diabetes can increase the risk of influenza infection, the severity of the disease, and the fatal consequences of this infection [52–55]. About 90% of patients with type 2 diabetes are overweight, and obesity is a significant risk factor for severe influenza infection [56]. This reinforcing effect of diabetes on influenza may be due to the inhibitory effect of hyperglycemia on the immune system [57–59]. It has been shown that this hyperglycemia-associated immunosuppression and susceptibility to influenza infection can be alleviated by insulin administration and diabetes control [60]. On the other hand, the pentose phosphate pathway (PPP), as another glucose-consuming pathway reported to be enhanced by IVI [61], contributes to a higher yield of nucleotides and ATP for viral replication [62]. Significant up-regulation of the PPP key enzymes in influenza-infected cells, including glucose 6-phosphate dehydrogenase (G6PD) and 6-phosphogluconate dehydrogenase (6PGD), was reported by Janke et al. [63]. G6PD enzyme is also responsible for the generation of NADPH [64], a critical component of fatty acid biosynthesis. The level of G6PD activity specifies the ability of the cell to clear the accumulated ROS. Cells with an average G6PD level can retain the appropriate GSH/GSSG ratio and keep the ROS production at a tolerably low level, indicating that G6PD activity has an inverse correlation with cellular ROS level [65]. Disruption of redox balance has been shown to contribute to replication and virulence of several viruses [66–68], and G6PD deficiency can cause this disruption. Despite the results reported by Janke et al., G6PD activity seems to have an inverse relation with some other respiratory viral infections. For example, in an in vitro study, after infection with human coronavirus (HCoV) 229E, the production of viral particles in G6PD-deficient or G6PD-knockdown cells was higher than in healthy cells, and this was correlated with increased oxidant production [67].

Molecular mechanisms through which the virus can control the metabolic pathways have been thoroughly identified. Smallwood et al. have shown that an increase in glucose uptake, glycolysis, and glutaminolysis following influenza infection may be related to the loss of PI3K/AKT/mTOR pathway homeostasis and subsequent increase in c-Myc expression in the infected cells [9]. Regarding the available results, the mechanistic target of rapamycin complex 1 (mTORC1) and mTORC2 signaling can be activated by a variety of influenza virus proteins. The viral hemagglutinin (HA) protein, along with virus replication, can upregulate PDPK1-mediated phosphorylation and activate AKT, which is required for induction of the mTORC1 signaling pathway by the influenza virus. On the other hand, influenza M2 protein is capable of down-regulation of the mTORC1 inhibitor REDD1, thereby enhancing the mTORC1 activation [69]. mTORC1 signaling, in turn, promotes c-Myc expression at the translational level [70]. Additionally, the NS1 protein can effectively promote the activity of mTORC2, which, in turn, upregulates c-Myc through FoxO inhibition [71]. Moreover, AKT-dependent inactivation of FoxOs can increase glycolysis [72, 73] by removing the suppressive force of c-Myc [74–76]. Myc enhances glycolysis by upregulating expression of the glucose transporter GLUT1, glycolytic genes, and lactate dehydrogenase (LDH), as the converter of pyruvate to lactate [77, 78]. mTORC1 also mediates upregulation of hypoxia-inducible factor-1α (HIF-1α), a factor that increases the expression of various genes [79], including several glycolytic enzymes, glucose transporters, and LDH [80–82]. AKT is able to promote the expression and membrane localization of GLUT1 as well as the function of phosphofructokinase [83, 84]. Furthermore, AKT is demonstrated to activate SREBP in an mTORC1-dependent manner [85] and to upregulate SREBP by enhancing the stability of its processed form [86]. SREBP1 is shown to be required for the mTORC1-induced increase in the expression of G6PD, which is the rate-limiting enzyme of the PPP oxidative branch [79].

**Fig. 1:**
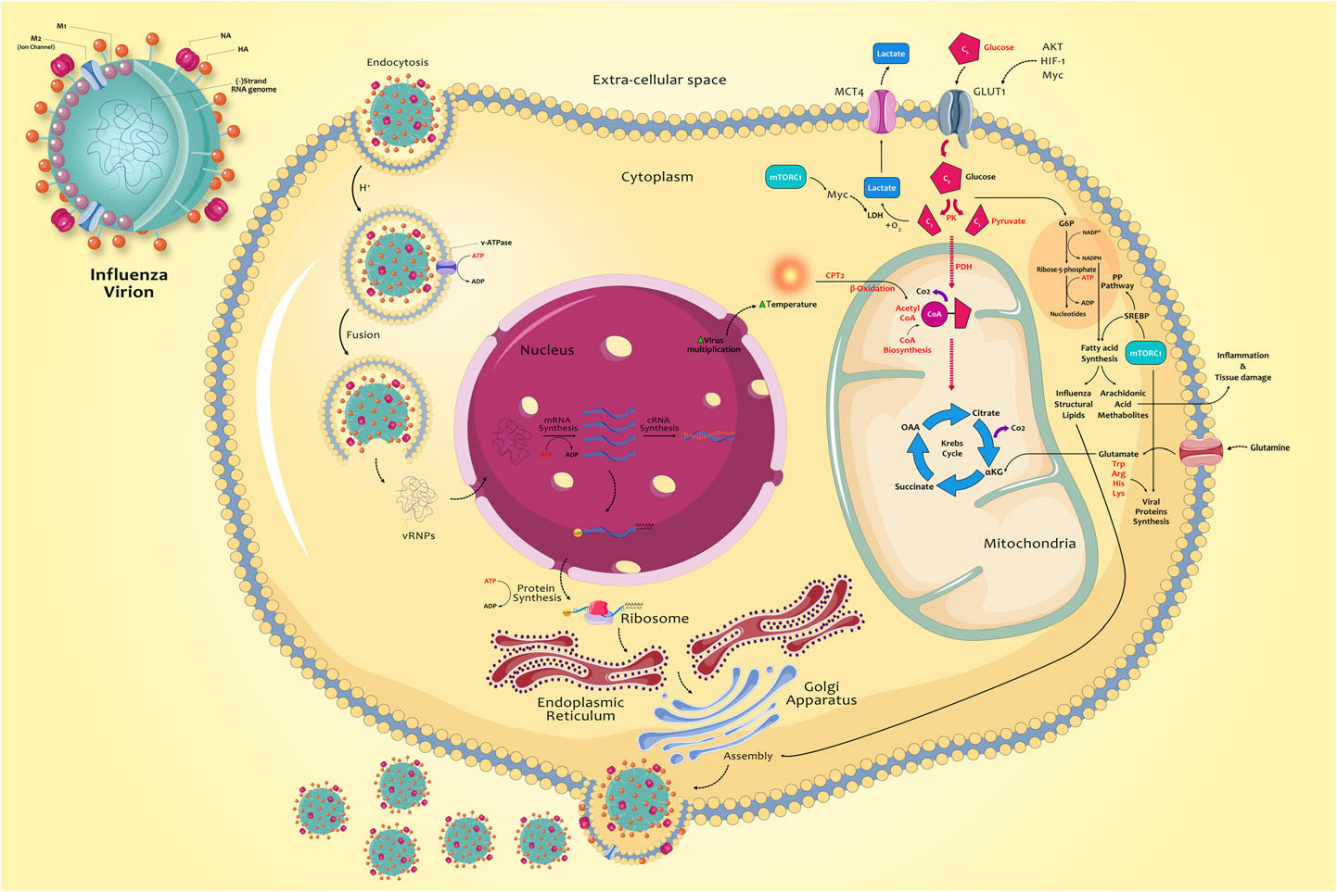
Metabolic changes caused by influenza infection and related mechanisms. Several anabolic and catabolic processes can be affected: higher glucose uptake and metabolism in glycolysis and pentose phosphate pathways, higher nucleotide catabolism, increase in biosynthesis of fatty acids including arachidonic acid, the precursor of proinflammatory lipids, and also enhanced glutaminolysis and protein synthesis. Activation of mTORC1&2 signaling and downstream factors by influenza infection may have an essential role in the upregulation of these metabolic processes. In addition, high ATP consumption and reduced β-oxidation, as well as glucose oxidation by influenza infection, contribute to the ATP crisis and hence influenza-related multi-organ failure

In addition to the above-mentioned metabolic pathways, the influenza virus exhibits disruptive effects on some other metabolic processes, which gives rise to metabolic disorders and ATP crisis. Previous studies have found that the influenza infection increases the cellular synthesis of fatty acids [87], with some of their derivatives, including eicosanoids [88], and these molecules are natural endogenous ligands and stimulators of peroxisome proliferation-activated receptors (PPARs) [88–90]. PPARs are a group of nuclear receptor proteins that act as transcription factors and regulate the expression of different genes [91] involved in cellular differentiation and metabolism of carbohydrates, lipids, and proteins [92]. Furthermore, all types of PPARs discovered so far are able to suppress the activity of the pyruvate dehydrogenase (PDH) enzyme (known as a catalyzer of the oxidative decarboxylation of pyruvate leading to acetyl-CoA production) in various organs through the upregulation of pyruvate dehydrogenase kinase (PDK)-4 [93]. In this respect, extremely low PDH enzyme activity has been found after influenza infection in vitro. Enhanced PDK4-mediated inhibition of PDH has been found in the lung tissue of influenza-infected mice. This enzyme inhibition contributes to an erroneous process, which causes significant disruption of glucose oxidation, cellular respiration, and lipid metabolism (Fig. 1) [7, 94].

### Lipid metabolism

Following infection, levels of phospholipids and several lipid derivatives undergo perturbations. Several lines of evidence have shown that in obese mice due to abnormalities in the metabolism of fatty acids and phospholipids, induction of influenza infection produces a more severe inflammatory response in comparison with non-obese mice [95]. These findings indicate a direct link between influenza-mediated inflammation and the abundance of lipids and their metabolism in the body. According to a study, diacyl glycerophosphocholine (PC) and diacyl glycerophosphoethanolamine (PE) species (containing 20:4 or 22:6 as precursors of docosahexaenoic acid (DHA) and arachidonic acid (AA) respectively) are associated with influenza-related diseases. Interestingly, glycerophospholipid species (PC (18:1/20:4) and PC (16:0/22:6)) yields increase during the influenza infection. In addition, upsurge in PC (18:1/20:4) and PE (18:0/22:4) species is associated with tissue lesions in the lungs and trachea. Several phospholipids containing 20:4, especially PC (18:1/20:4), serve as AA reservoirs in cells, breakdown of which increases the cellular levels of AA [96]. This influenza-mediated elevation of AA, consistent with inflammation, has also been reported in the same study [97]. The accumulation of the above-mentioned proinflammatory lipids in the cell leads to the promoted synthesis of eicosanoids and inflammatory mediators, thus exacerbating post-infection necrosis, inflammation, and tissue damage in the lungs [96]. In a prospective cohort study on influenza-infected subjects, lipid inflammatory mediators in serum samples of patients were mainly AA-derived oxylipins, including TXB2, 15-deoxy-12,14-PGD2, 20-HETE, 5,6-DHET, 5-oxoETE, LTE4, and 12-HpETE. Although all of these metabolites were shown to be elevated shortly after the infection, 5,6-DHET and 5-oxoETE levels remained considerably high up to 4 weeks post-infection, indicating a constant pulmonary inflammation [98].

The pulmonary surfactant system, which is involved in suppressing influenza infection in the respiratory tracts [99], can be disrupted due to significant influenza-induced changes in the abundance of different types of PC and PE species as the major components of surfactants [96, 100]. Tanner et al. proposed a principal correlation between influenza replication and choline lipids metabolism. They found an IVI-mediated reduction in ester-linked PC species as well as an increased level of sphingomyelin (SM) [101], probably connected with expending cellular choline stores for SM synthesis. This led to an increase in SM species within the infected cell. SM and short-chain fatty acid-containing ether-linked PC (ePC) species were found in higher amounts in both infected cells and virions and therefore appeared to be involved in viral morphogenesis. On the other hand, long-chain fatty acid-containing ePC was increased in infected cells while having low levels in the structure of the virion, highlighting the role of this phospholipid in replication of the virus [101].

Evidently, higher production of these complex lipids in the cell will require increased biosynthesis of fatty acids. In this regard, the results of a study revealed that influenza infection could induce fatty acid biosynthesis and cholesterol metabolism in human lung basal epithelial tumor cells [87]. Since SREBPs are thoroughly identified as stimulators of expression of many genes involved in lipid and sterol biosynthesis, including fatty acid synthase [102–104], their upregulation by the influenza virus (through induction of mTORC1 signaling, as discussed earlier) may logically explain the increased rate of lipogenesis [105]. A coincidence between increased fatty acid synthesis and a decline in fatty acid β-oxidation has been found during influenza infection, which is attributed to a variety of mechanisms directly or indirectly related to viral replication. For instance, the sharp increase of proinflammatory cytokines during influenza infection [106] causes decreased hepatic fatty acid β-oxidation both in vitro and in vivo [107, 108], most likely through excessive nitric oxide and other related free radicals [109]. In addition, increased temperature of cells during infection (which could be the result of virus replication and fever) causes heat stress which in turn can considerably downregulate carnitine palmitoyltransferase II (CPT II) activity and reduce the β-oxidation and ATP levels in fibroblasts of influenza-associated encephalopathy patients and healthy volunteers [110]. A study on influenza-infected mice demonstrated a significant depression in long hepatic chain fatty acid β-oxidation at both the mRNA and protein level, as several β-oxidation essential enzymes were reduced by > 50% [97]. A significant decrease in mitochondrial fatty acid β-oxidation simultaneously with increased biosynthesis of fatty acids and membrane lipids may reflect the fact that the virus stores structural lipids to produce more infectious particles. In addition to impaired metabolism in mitochondria, influenza infection induces severe peroxisomal lipid metabolism disorders, which can be inferred from abnormal levels of several specific long-chain fatty acids [101] (Fig. 1).

### Other metabolites

The aforementioned metabolic processes are not the only pathways affected by influenza virus infection. This virus has the ability to induce higher consumption rates of glutamine during glutaminolysis, which can be attributed to transient c-Myc overexpression [9]. Myc acts to regulate glutamine uptake and its utilization in the cell [111]. It has been demonstrated that catalytic activity of glutaminase, as the key enzyme in glutaminolysis, greatly increases following the infection [63]. Moreover, essential amino acids, especially tryptophan, are other materials whose quantities have been shown to decline in infected tissues [112]. mTORC1 can up-regulate protein synthesis through several downstream factors [113]. Thus, induction of mTORC1 signaling by the influenza virus leads to higher usage of essential amino acid storages for concurrent production of large amounts of viral and cellular proteins. Infection of influenza virus can also alter the cellular level and metabolism of purines and pyrimidines [8, 98, 100], and is associated with both increased activities of nucleotide catabolism core enzymes including adenosine deaminase (ADA) and xanthine oxidase (XO) and elevated levels of inosine, hypoxanthine, xanthine, and uric acid in serum and bronchoalveolar lavage fluid. Enhanced catabolic degradation of nucleotides and their metabolites can facilitate the production of superoxide and contribute to the pathogenesis of influenza infection [23].

### Immunometabolites and their role in influenza infection

Interferons are well-known cytokines with a powerful capability of altering the cellular functions following viral infections. These alterations affect protein synthesis, composition of the membrane, cellular proliferation, and nutritional status [34]. Interferon stimulated genes (ISGs) are the effector components whose transcription could be induced by type I IFNs and IFNγ [114, 115].

### IFNs and energy metabolism

Studies have recently underscored the general effect of IFNs on the energy metabolism of cells, mostly by promoting glycolysis. For instance, IFNβ has been shown to induce the glucose uptake of embryonic fibroblasts in a PI3/AKT-dependent manner, thereby increasing ATP production [116]. It has also been demonstrated that type I IFN can stimulate oxygen consumption in a range of cells, including conventional dendritic cells (DCs), keratinocytes, and memory T cells [117]. Indeed, the high yield of ATP and mitochondrial fitness guarantee the host cell’s need for energy in plasmacytoid DCs (pDCs) and non-hematopoietic cells following challenges with viral pathogens [118]. These studies emphasized the mediatory effect of type I IFN on glycolysis induction via IFNAR1, Tyk2, and STAT1. It has also been shown that influenza infection stimulates pDCs to enhance their glycolysis and develop a Warburg-like remodeling of energy metabolism. This enhanced glycolysis leads to higher IFN production and, consequently, more potent antiviral activity [118]. IFNγ induces metabolic reprogramming of M1 macrophages as a rapid increase in aerobic glycolysis, followed by a reduction in oxidative phosphorylation. This metabolic reprogramming maintains cell viability and the inflammatory response while reducing dependence on mitochondrial oxidative metabolism. Excessive production of pro-inflammatory cytokines and chemokines in human monocytes/macrophages can be blocked by inhibition of aerobic glycolysis [119]. Also, activation of macrophages by IFNγ induces expression of the ATP-citrate lyase enzyme (ACLY), and blockage of ACLY activity reduces the production of ROS and nitric oxide [120].

**Fig. 2:**
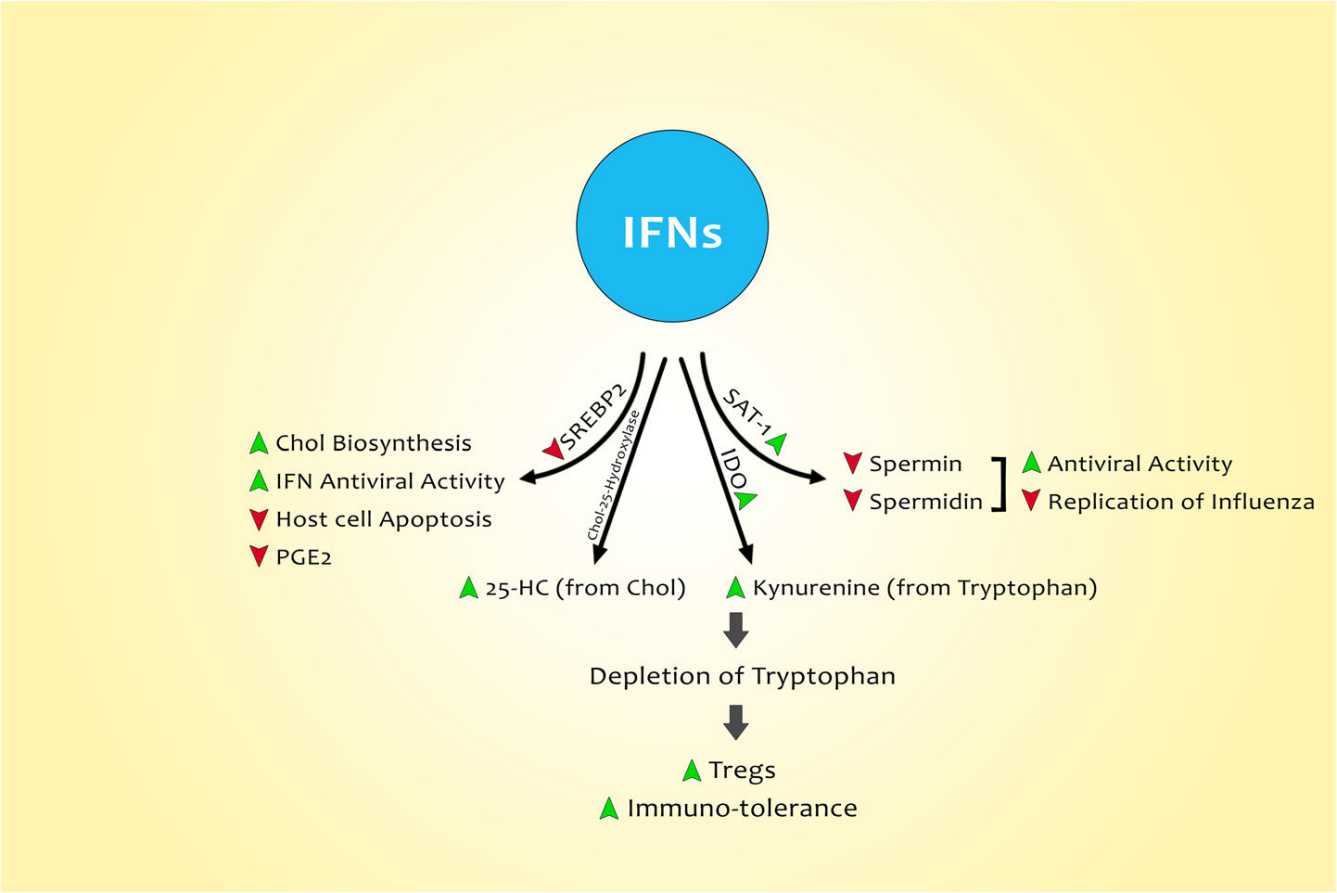
Role of IFNs in host cell metabolic changes following infection with influenza. IFNs affect the lipid metabolism through downregulation of SREBP-2, leading to higher cholesterol biosynthesis, and suppressed host cell apoptosis and PGE-2 production. They can also enhance the level of 25-HC arisen from cholesterol (via activation of Chol-25 hydroxylase) and IDO. SAT-1 can be upregulated by IFNs, lowering the levels of spermidine and spermine and thereby dampening influenza replication

There is a strong consensus that influenza replication is crucially dependent on fatty acids [97], which makes it a fascinating target for therapeutic modalities [45]. Thus, the ability of IFN to channel the FAs from biosynthesis to catabolism via fatty acid oxidation (FAO) is currently known as a promising antiviral strategy in pDCs [117], which requires further research for more elucidation. Several lines of current evidence have revealed the antiviral activity of type I IFN to be exerted through hampering glucose-derived cholesterol and fatty acid synthesis [121, 122]. Sterol regulatory binding protein 2 (SREBP2), along with SREBP1, is known as the leading transcription factor which orchestrates the biosynthesis pathway of sterol, whose inhibited transcription and expression can be strongly mediated by IFNs via IFNAR1 [123] (Fig. 2).

Innate immune cells can recognize influenza A viruses and their infected cells by toll-like receptors (TLRs) [124]. This recognition can lead to the induction of an inflammatory response that, in turn, controls the replication and spread of the virus [31]. H5N1, H7N7, and H7N9 were correlated with increased transcription of the cytokine response in mice. Severe infection with H7N9, H7N7, H5N1, or 1918 virus can lead to upregulation of inflammatory cytokine genes along with downregulation of lipid metabolism and coagulation genes [125]. This uncontrolled proinflammatory response accompanied by an inadequate anti-inflammatory response is referred to as the cytokine storm [31]. Monocytes/macrophages, neutrophils, and lung epithelial cells have useful roles in the cytokine storm developed by influenza infection [126]. Severe cytokine storm, with greater levels of interferons and tumor necrosis factors, has been recognized in patients hospitalized due to influenza infection [127]. Such influenza-induced cytokine storms, together with viral virulence, can develop severe lung injury in patients [128, 129]. It is believed that the level of the cytokine storm is directly associated with the severity of the disease caused by influenza infection [130, 131]. Some specific polymorphisms in immune system genes have determinative roles in the outcome of influenza infection. Our previous studies have shown a relationship between cytokine gene polymorphisms and severity of the influenza disease. Several cytokines were evaluated after influenza A/H3N2 virus infection, among which IL-17 rs2275913 GG and AG, GG and GT of IL-10 (rs1800872) and IL-28 (rs8099917) genotype TT polymorphisms were associated with increased risk of influenza infection.

In contrast, IL-1β (rs16944) (GG) and IL-28 (rs8099917) GG and TG genotypes were associated with reduced risk of infection [132]. In another study, an association between IL-1β rs16944 and IL-17 rs2275913 genotypes and severe influenza disease was found while IL-10 rs1800872 and IL-28 rs8099917 polymorphisms were not associated with influenza disease. Also, lacking an A allele in IL-17 rs2275913 could increase the risk of influenza A (H1N1) infection [2]. Such polymorphisms in immune system genes may be associated with some metabolic changes and, in turn, may reinforce the metabolic disorders following influenza infection. However, additional studies are needed in this field to confirm or reject this opinion.

### IFNs and amino acid metabolism and the role of IDO

IFNs are known to be capable of depletion of polyamines to limit virus replication. Polyamines are small ornithine-derived polycationic molecules which encompass three molecules: putrescine, spermidine, and spermine. Spermidine and spermine depletion is one of the compelling mechanisms through which IFNs produce their antiviral effects on the replication of RNA viruses. Mechanistically speaking, polyamines appear to play a pivotal role in the processes of RNA transcription and protein translation of viruses, making them a promising target to combat viral infections [133].

L-tryptophan is one of the nine essential amino acids with a remarkable role in immunosuppression and tolerance and is also essential in protein, kynurenine, and serotonin synthesis [122]. IDO is an intracellular enzyme that induces production of kynurenine from L-tryptophan, thereby acting to deplete tryptophan and modulate the immune system following viral infections [134]. Having two IFN-stimulated response elements and three IFNγ-activated sites in the promoter of IDO, IFNγ acts as the most powerful inducer of IDO1 expression [135]. IDO has also been shown to be expressed during influenza infection [136]. Dendritic cells, macrophages, and epithelial cells can express IDO [137, 138], and since the primary target for replication of influenza is primarily found to be respiratory epithelial cells, understanding the role of IDO during influenza infection is of particular importance. There exists a coincidence of peak IDO1 and IFN-k expression during influenza infection. Also, mouse lung airways considerably express IFN type I and III following infection with influenza [139]. These findings emphasize that there is upregulated expression and enhanced function of IDO during influenza infection, which is found to be induced by IFN-I. Moreover, IFN-I is thought to signal the adjacent cells via IFN-IR and stimulate them to produce IDO [140].

Nonetheless, the IFN-mediated IDO induction during influenza infection generally has undesirable consequences and establishes immune tolerance [136]. Indeed, an inhibitory effect of tryptophan depletion on T cell responses has been confirmed. Also, IDO induces kynurenine derivation from tryptophan, leading to stimulation of regulatory T cells [141].

**Fig. 3:**
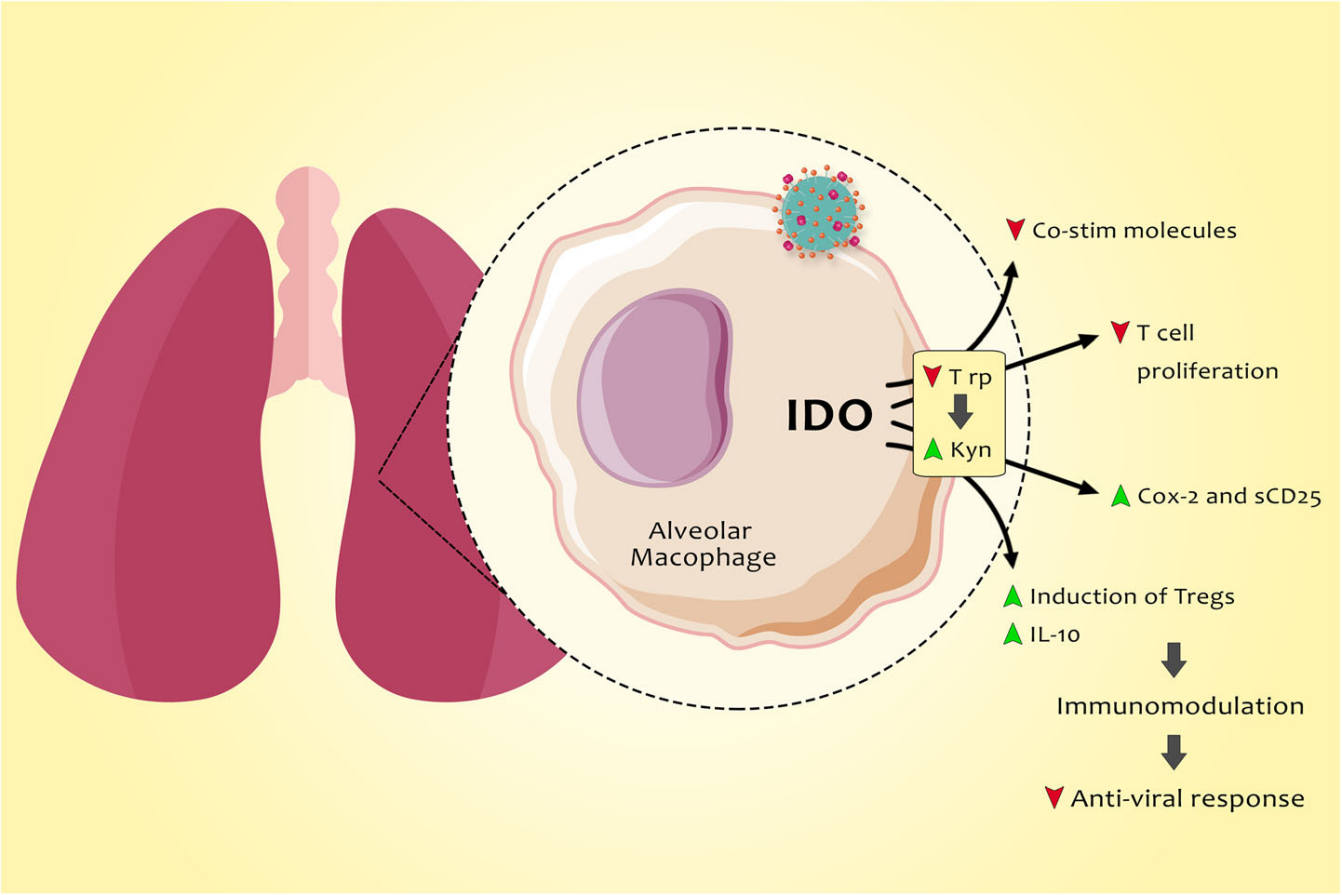
Role of IFN in IDO activation. IFNs also induce activity of IDO, an enzyme that mediates the production of kynurenine from tryptophan. This event results in tryptophan depletion, which in turn develops the immune tolerance and generates Tregs

Nowadays, IDO is hypothesized to be part of the “metabolic, immune regulation,” which plays a protective role in immune responses and inhibits the overreaction of these responses against influenza infection. A pleiotropic role has been attributed to IDO during infections, which gives rise to the opposing outcomes (Fig. 3) [142].

Research on the role of IDO in influenza infection has been mainly focused on the murine models of influenza infection, emphasizing the increased IDO activity and its maximum expression correlated with increased lymphocyte numbers in the respiratory tract [143].

### IFNs and nitric oxide induction

Nitric oxide (NO) is a gaseous free radical with accessible vasodilatory and microbicidal functions [144]. However, the antiviral effect of NO has also been documented, leading to reduced viral load and more efficient clearance of infection [145]. Nitrosylation of viral molecules has been offered as an antiviral mechanism employed by NO [146]. Also, NO synthase-mediated generation of NO leads to the depletion of L-arginine, thereby reducing the level of polyamines. Thus, IFN-induced NOS2 represents antiviral activities, and this, in turn, may exhibit another mechanism through which IFNs hinder viral infections such as influenza [34]. Despite existing data regarding the antiviral activity of NO, many studies have considered NO as a double-edged sword with both pathogenic and viricidal effects. The role of NO in the pathogenesis of pneumonia caused by influenza virus infection has been described in mice. The IFNγ response induces greatly increased levels of iNOS in the lungs of infected mice, leading to the production of a significant amount of NO and peroxynitrite species, which are among the most important pathogenic factors in influenza virus-induced pneumonia in mice [147]. Uetani et al. also observed overexpression of the iNOS gene in human airway epithelial cells induced by influenza A virus infection [148]. In addition, NO produced by phagocytic cells has antiviral activity that is simultaneous with nonspecific damage of host cells and viral pathogenesis [149]. In a survey by Nin et al. on pandemic A/H1N1 influenza infection, all cases showed increased levels of iNOS protein, tyrosine nitration, and oxygen free radicals, indicating the production of peroxynitrite. Their results revealed the involvement of oxidative and nitrative stress in the pathogenesis of H1N1 influenza virus-induced acute respiratory distress syndrome (ARDS) [15]. Influenza-induced cytokines such as IFNγ stimulate NO release from human airway epithelial cells [150–152]. As mentioned previously, the influenza infection induces upregulation of HIF-1α.

Interestingly HIF-1α-knockout macrophages show decreased expression of iNOS after IFNγ stimulation [153], indicating the possible involvement of HIF-1α in influenza pathogenesis. It has been shown that infection of H5N1 and 1918 viruses induces higher levels of NO in mice compared to the seasonal H1N1 virus and, as a result, they develop more intense pathogenic outcomes, while mice with iNOS deficiency showed reduced morbidity, mortality, and cytokine production in the lungs following H5N1 and 1918 virus infection. Also, systemic administration of NOS inhibitor could postpone weight loss and death among 1918 virus-infected mice [154]. In another survey, the delivery of NO to influenza-infected mice could not improve the lung infection and survival of mice, indicating that NO administration was not a suitable treatment strategy for influenza although this was probably due to the difficulty of determining concentrations of NO that are both viricidal and safe in host airways [155]. Also, it is reported that NO released from *S*-nitroso-*N*-acetylpenicillamine (SNAP) reduces the replication of influenza virus in a dose-dependent manner. The production of NO in airway epithelial cells can lead to antiviral rather than harmful effects following influenza infection provided that its production is precisely controlled [156].

### Novel therapeutic approaches by targeting metabolic pathways

Since the influenza virus affects about 20% of the world population annually, preventive and therapeutic approaches require much closer attention. Therapeutic drugs for influenza infection fall into three groups: 1) neuraminidase inhibitors (zanamivir, oseltamivir, laninamivir, peramivir), 2) M2 inhibitors (rimantadine and amantadine), and 3) polymerase inhibitors (favipiravir) [157, 158]. Antiviral drug resistance has recently emerged as a global problem which can bring about a remarkable financial and social burden [159]. Therefore, further research is urgently needed to develop novel and promising antiviral drugs. Many of the metabolic pathways in influenza infections are increasingly changing, dampening of which appears to hamper the virus replication. One of the newly developed strategies aiming to hinder influenza infection is targeting metabolic pathways and restoration of hemostasis in cells (Table 1).

**Table 1:** Characteristics of some metabolic pathway blockers in influenza infection

Agent	Target	Outcome	Model	Reference
BEZ235	PI3K/mTOR	Reconstitution of metabolic status and decreased viral replication	In vivo	[9]
Hochuekkito	Effect on mitochondrial and glycolysis	Ameliorates metabolism and intensifies the symptoms	In vitro	[160]
Simvastatin	Sterol synthesis	Decreased influenza replication and cytokine production	In vitro	[161]
DADA	Pyruvate dehydrogenase kinase	Suppresses cytokine storm and viral replication	In vivo	[94]
MJWQH	amino acid, fatty acid and arachidonic acid pathway	Improved weight loss, lung index, biomarkers and inflammatory mediators such as prostaglandin E2	In vivo	[162]
Bezafibrate	Carnitine palmitoyltransferase II	Restores the ATP levels in cells and intensifies the symptoms	In vivo	[110]
AM580	Sterol regulatory element binding protein (SREBP) pathways	Inhibited influenza virus replication through interference with SREBP paths	In vivo	[105]

*DADA* Diisopropylamine dichloroacetate, *MJWQH* Modified Jiu Wei Qiang Huo

The PI3K/mTOR signaling pathway has been shown to play a pivotal role in a variety of cellular pathways, including proliferation and nutrient uptake, and its activation increases the glucose uptake through the up-regulation of cell surface glucose transporter [163]. BEZ235 alters glucose metabolism via blockage of the PI3K/mTOR pathway, and some clinical trials are underway to assess this strategy in cancer therapy (Smith et al., 2012). On the other hand, several lines of evidence have demonstrated that siRNA targets the PI3K-AKT-mTOR pathway, thereby warding off influenza infection [164]. In a new study by Smallwood et al., it was found that although BEZ235 did not interfere with the early stages of the infection, it could finally reduce the viral progeny and result in prolonged survival in mice challenged by the influenza virus. Indeed, BEZ235 induced hemostasis in the PI3K/mTOR pathway via phosphorylation of p85 and 4E-BP1 and through reconstitution of metabolic status, which was already altered by the virus [9].

It has been found that there is an elevated level of PDK4 in lung, liver, and heart during influenza infection, while the levels of ATP and PDH, a key enzyme in the regulation of glucose, lipid and ATP levels in human cells, are shown to be reduced [156]. Furthermore, dichloroacetate (DCA) is a pyruvate dehydrogenase kinase inhibitor with anti-tumor activity in a variety of carcinomas. Studies have also indicated that diisopropylamine dichloroacetate (DADA) could ameliorate the metabolism of hepatocytes in chronic liver disease [165]. In a study by Yamane et al. attempting to evaluate the effect of DADA on influenza-infected mice (PR8), oral administration of DADA was found to not only restore the activity of PDH and ATP in affected organs but also suppress cytokine storm and viral replication [94].

Sterols are intermediate metabolites that play an essential role in a broad spectrum of biological pathways, including inflammation. Research has shown that interferon production following the immune response in viral infection regulates sterol production paths. Blanc et al. revealed that sterol metabolism pathway regulators such as simvastatin, Zometa (zoledronic acid), and FPT inhibitor III could effectively hinder H5N1 influenza replication and cytokine production, which makes them promising therapeutic candidates in acute patients [121, 161].

On the other hand, as mentioned earlier, SREBPs are transcription factors that have a critical role in the process of lipogenesis. Studies have shown that these factors can play a variety of roles, such as energy supply and post-translational protein modification, as well as in the propagation of various groups of viruses such as influenza viruses. A study has shown that the AM580 compound, which is a retinoid derivative, inhibits SREBP-linked pathways, and it has antiviral activity against influenza A and coronavirus in vitro and in vivo [105].

Concerning the fact that mitochondria and glycolysis are two sources of energy production, they play vital roles in the regulation of innate immunity responses. During the immune system response, and especially the cytokine storm, following influenza infection, ATP synthesis in the mitochondria decreases, leading to weakened innate immune responses (Dengbing Yao). Studies have revealed that traditional herbal medicines have an important role in improving influenza-like symptoms in infected patients. Results of a study demonstrated that pre-treatment of infected cells with Hochuekkito (a traditional Japanese herbal medicine) could activate both mitochondrial and glycolytic energy metabolism and thereby intensify symptoms [160]. Also, the effects of traditional Chinese medicine (modified Jiu Wei Qiang Huo) on H1N1 infected mice were evaluated in another study. The results showed that this herbal medicine could ameliorate weight loss and inflammatory mediators in infected mice through the regulation of amino acid, fatty acid, and arachidonic acid pathways [162].

## Conclusion

Based on recent studies, influenza virus infection can interfere with cellular metabolic pathways either directly or indirectly via stimulation of immune system mediators. Through enhancing the activity of the mTORC1 complex, the influenza virus strengthens several metabolic pathways, including glycolysis, glutaminolysis, pentose phosphate, and fatty acid synthesis, to provide more ATP and structural materials for viral replication. On the other hand, β-oxidation suppression following viral infection can help to supply essential fatty acids for the synthesis of structural lipids. However, exhausting cellular ATP resources due to virus replication, as well as an increase in pro-inflammatory lipid synthesis, will ultimately lead to irreversible cell damage. Innate immune responses following influenza infection play a crucial role in metabolic alterations. IFN is one of these mediators that acts on several metabolites such as IDO and NO and thereby affects lipid and amino acid pathways. Since the drug resistance in influenza infection is a global concern, research on designing novel therapeutic modalities to tackle pandemics is of particular importance. Thus, a clear understanding of the metabolic alterations during influenza infection would be tremendously helpful for therapeutic purposes.

## Data Availability

Not applicable.

## Abbreviations

6PGD: 6-phosphogluconate dehydrogenase
AA: Arachidonic acid
ADA: Adenosine deaminase
CPT: Carnitine palmitoyltransferase
DADA: Diisopropylamine dichloroacetate
DCA: Dichloroacetate
DHA: Docosahexaenoic acid
FA: Fatty acid
FAO: Fatty acid oxidation
IDO: Indoleamine-2,3-dioxygenase
ISGs: Interferon stimulated genes
IVI: Influenza virus infection
MOI: Multiplicity of infection
mTORC1: Mechanistic target of rapamycin complex 1
NHBE: Normal human bronchial epithelial
NO: Nitric oxide
PDH: Pyruvate dehydrogenase
PDK: Pyruvate dehydrogenase kinase
PPARs: Peroxisome proliferation-activated receptors
PPP: Pentose phosphate pathway
SM: Sphingomyelin
SREBP2: Sterol regulatory binding protein 2
XO: Xanthine oxidase

## References

[1] Keshavarz M, Namdari H, Arjeini Y, Mirzaei H, Salimi V, Sadeghi A, Mokhtari-Azad T, Rezaei F (2019). Induction of protective immune response to intranasal administration of influenza virus-like particles in a mouse model. J Cell Physiol.

[2] Keshavarz M, Namdari H, Farahmand M, Mehrbod P, Mokhtari-Azad T, Rezaei F (2019). Association of polymorphisms in inflammatory cytokines encoding genes with severe cases of influenza a/H1N1 and B in an Iranian population. Virol J.

[3] Mizuguchi M, Yamanouchi H, Ichiyama T, Shiomi M (2007). Acute encephalopathy associated with influenza and other viral infections. Acta Neurol Scand.

[4] Sanei F, Wilkinson T (2016). Influenza vaccination for patients with chronic obstructive pulmonary disease: understanding immunogenicity, efficacy and effectiveness. Ther Adv Respir Dis.

[5] Vasileiou E, Sheikh A, Butler C, El Ferkh K, Von Wissmann B, McMenamin J, Ritchie L, Schwarze J, Papadopoulos NG, Johnston SL (2017). Effectiveness of influenza vaccines in asthma: a systematic review and meta-analysis. Clin Infect Dis.

[6] Eagle H, Habel K (1956). The nutritional requirements for the propagation of poliomyelitis virus by the HeLa cell. J Exp Med.

[7] Kido H, Indalao IL, Kim H, Kimoto T, Sakai S, Takahashi E (2016). Energy metabolic disorder is a major risk factor in severe influenza virus infection: proposals for new therapeutic options based on animal model experiments. Respir Investig.

[8] Chandler JD, Hu X, Ko E-J, Park S, Lee Y-T, Orr M, Fernandes J, Uppal K, Kang S-M, Jones DP, Go YM (2016). Metabolic pathways of lung inflammation revealed by high-resolution metabolomics (HRM) of H1N1 influenza virus infection in mice. Am J Phys Regul Integr Comp Phys.

[9] Smallwood HS, Duan S, Morfouace M, Rezinciuc S, Shulkin BL, Shelat A, Zink EE, Milasta S, Bajracharya R, Oluwaseum AJ (2017). Targeting metabolic reprogramming by influenza infection for therapeutic intervention. Cell Rep.

[10] Lazrak A, Iles KE, Liu G, Noah DL, Noah JW, Matalon S (2009). Influenza virus M2 protein inhibits epithelial sodium channels by increasing reactive oxygen species. FASEB J.

[11] Shin N, Pyo C-W, Jung KI, Choi S-Y (2015). Influenza a virus PB1-F2 is involved in regulation of cellular redox state in alveolar epithelial cells. Biochem Biophys Res Commun.

[12] Lim J, Oh E, Kim Y, Jung W, Kim H, Lee J, Sul D (2014). Enhanced oxidative damage to DNA, lipids, and proteins and levels of some antioxidant enzymes, cytokines, and heat shock proteins in patients infected with influenza H1N1 virus. Acta Virol.

[13] Ng MP, Lee JC, Loke WM, Yeo LL, Quek AM, Lim EC, Halliwell B, Seet RC-S (2014). Does influenza A infection increase oxidative damage?.

[14] Erkekoğlu P, Aşçı A, Ceyhan M, Kızılgün M, Schweizer U, Ataş C, Kara A, Koçer-Giray B. Selenium levels, selenoenzyme activities and oxidant/antioxidant parameters in H1N1-infected children. Turk J Pediatr. 2013;55:271–82. 24217073

[15] Nin N, Sanchez-Rodriguez C, Ver L, Cardinal P, Ferruelo A, Soto L, Deicas A, Campos N, Rocha O, Ceraso D (2012). Lung histopathological findings in fatal pandemic influenza a (H1N1). Med Int.

[16] Amatore D, Sgarbanti R, Aquilano K, Baldelli S, Limongi D, Civitelli L, Nencioni L, Garaci E, Ciriolo MR, Palamara AT (2015). Influenza virus replication in lung epithelial cells depends on redox-sensitive pathways activated by NOX4-derived ROS. Cell Microbiol.

[17] Buffinton G, Christen S, Peterhans E, Stocker R (1992). Oxidative stress in lungs of mice infected with influenza a virus. Free Radic Res Commun.

[18] Hennet T, Peterhans E, Stocker R (1992). Alterations in antioxidant defences in lung and liver of mice infected with influenza a virus. J Gen Virol.

[19] Ye S, Lowther S, Stambas J (2015). Inhibition of reactive oxygen species production ameliorates inflammation induced by influenza a viruses via upregulation of SOCS1 and SOCS3. J Virol.

[20] Vlahos R, Stambas J, Bozinovski S, Broughton BR, Drummond GR, Selemidis S (2011). Inhibition of Nox2 oxidase activity ameliorates influenza a virus-induced lung inflammation. PLoS Pathog.

[21] To E, Broughton BR, Hendricks KS, Vlahos R, Selemidis S (2014). Influenza a virus and TLR7 activation potentiate NOX2 oxidase-dependent ROS production in macrophages. Free Radic Res.

[22] Vlahos R, Luong R, Halls ML, Reading PC, King PT, Chan C, Drummond GR, Sobey CG, Broughton BR, To EE (2017). Endosomal NOX2 oxidase exacerbates virus pathogenicity and is a target for antiviral therapy. Nat Commun.

[23] Akaike T, Ando M, Oda T, Doi T, Ijiri S, Araki S, Maeda H (1990). Dependence on O2-generation by xanthine oxidase of pathogenesis of influenza virus infection in mice. J Clin Invest.

[24] Oda T, Akaike T, Hamamoto T, Suzuki F, Hirano T, Maeda H (1989). Oxygen radicals in influenza-induced pathogenesis and treatment with pyran polymer-conjugated SOD. Science.

[25] Pyo C-W, Shin N, Jung KI, Choi JH, Choi S-Y (2014). Alteration of copper–zinc superoxide dismutase 1 expression by influenza a virus is correlated with virus replication. Biochem Biophys Res Commun.

[26] Cai J, Chen Y, Seth S, Furukawa S, Compans RW, Jones DP (2003). Inhibition of influenza infection by glutathione. Free Radic Biol Med.

[27] Nencioni L, Iuvara A, Aquilano K, Ciriolo MR, Cozzolino F, Rotilio G, Garaci E, Palamara AT (2003). Influenza a virus replication is dependent on an antioxidant pathway that involves GSH and Bcl-2. FASEB J.

[28] Celestino I, Checconi P, Amatore D, Coluccio P, Dattilo R, Alunni Fegatelli D, Clemente AM, Torcia MG, Matarrese P, Mancinelli R (2018). Differential redox state contributes to sex disparities in the response to influenza virus infection in male and female mice. Front Immunol.

[29] Amatore D, Celestino I, Brundu S, Galluzzi L, Coluccio P, Checconi P, Magnani M, Palamara AT, Fraternale A, Nencioni L (2019). Glutathione increase by the n-butanoyl glutathione derivative (GSH-C4) inhibits viral replication and induces a predominant Th1 immune profile in old mice infected with influenza virus. FASEB BioAdv.

[30] Alam K, Ghousunnissa S, Nair S, Valluri VL, Mukhopadhyay S (2010). Glutathione-redox balance regulates c-rel–driven IL-12 production in macrophages: possible implications in Antituberculosis immunotherapy. J Immunol.

[31] Liu Q, Zhou Y-H, Yang Z-Q (2016). The cytokine storm of severe influenza and development of immunomodulatory therapy. Cell Mol Immunol.

[32] Coates Bria M., Staricha Kelly L., Koch Clarissa M., Cheng Yuan, Shumaker Dale K., Budinger G. R. Scott, Perlman Harris, Misharin Alexander V., Ridge Karen M. (2018). Inflammatory Monocytes Drive Influenza A Virus–Mediated Lung Injury in Juvenile Mice. The Journal of Immunology.

[33] Adams O, Besken K, Oberdörfer C, MacKenzie C, Takikawa O, Däubener W (2004). Role of indoleamine-2, 3-dioxygenase in alpha/beta and gamma interferon-mediated antiviral effects against herpes simplex virus infections. J Virol.

[34] Fritsch SD, Weichhart T (2016). Effects of interferons and viruses on metabolism. Front Immunol.

[35] Munger J, Bajad SU, Coller HA, Shenk T, Rabinowitz JD (2006). Dynamics of the cellular metabolome during human cytomegalovirus infection. PLoS Pathog.

[36] Munger J, Bennett BD, Parikh A, Feng XJ, McArdle J, Rabitz HA, Shenk T, Rabinowitz JD (2008). Systems-level metabolic flux profiling identifies fatty acid synthesis as a target for antiviral therapy. Nat Biotechnol.

[37] Landini MP (1984). Early enhanced glucose uptake in human cytomegalovirus-infected cells. J Gen Virol.

[38] Bardeletti G (1977). Respiration and ATP level in BHK21/13S cells during the earlist stages of rubella virus replication. Intervirology.

[39] Bardeletti G, Henry M, Sohier R, Gautheron D (1972). Primary effects of the rubella virus on the metabolism of BHK-21 cells grown in suspension cultures. Arch Gesamte Virusforsch.

[40] El-Bacha Tatiana, Midlej Victor, Pereira da Silva Ana Paula, Silva da Costa Leandro, Benchimol Marlene, Galina Antonio, Da Poian Andrea T. (2007). Mitochondrial and bioenergetic dysfunction in human hepatic cells infected with dengue 2 virus. Biochimica et Biophysica Acta (BBA) - Molecular Basis of Disease.

[41] Green M, Henle G, Deinhardt F (1958). Respiration and glycolysis of human cells grown in tissue culture. Virology.

[42] Baron S, Levy HB (1956). Some metabolic effects of poliomyelitis virus on tissue culture. Nature.

[43] Levy HB, Baron S (1957). The effect of animal viruses on host cell metabolism. II. Effect of poliomyelitis virus on glycolysis and uptake of glycine by monkey kidney tissue cultures. J Infect Dis.

[44] Burgener A, Coombs K, Butler M (2006). Intracellular ATP and total adenylate concentrations are critical predictors of reovirus productivity from Vero cells. Biotechnol Bioeng.

[45] Sanchez EL, Lagunoff M (2015). Viral activation of cellular metabolism. Virology.

[46] Gatenby RA, Gillies RJ (2004). Why do cancers have high aerobic glycolysis?. Nat Rev Cancer.

[47] Ritter JB, Wahl AS, Freund S, Genzel Y, Reichl U (2010). Metabolic effects of influenza virus infection in cultured animal cells: intra-and extracellular metabolite profiling. BMC Syst Biol.

[48] Petiot E, Jacob D, Lanthier S, Lohr V, Ansorge S, Kamen AA (2011). Metabolic and kinetic analyses of influenza production in perfusion HEK293 cell culture. BMC Biotechnol.

[49] Genzel Y, Behrendt I, Konig S, Sann H, Reichl U (2004). Metabolism of MDCK cells during cell growth and influenza virus production in large-scale microcarrier culture. Vaccine.

[50] Kohio HP, Adamson AL (2013). Glycolytic control of vacuolar-type ATPase activity: a mechanism to regulate influenza viral infection. Virology.

[51] Maruyama H, Kimura T, Liu H, Ohtsuki S, Miyake Y, Isogai M, Arai F, Honda A (2018). Influenza virus replication raises the temperature of cells. Virus Res.

[52] Diepersloot R, Bouter KP, Beyer W, Hoekstra J, Masurel N (1987). Humoral immune response and delayed type hypersensitivity to influenza vaccine in patients with diabetes mellitus. Diabetologia.

[53] Valdez R, Narayan K, Geiss LS, Engelgau MM (1999). Impact of diabetes mellitus on mortality associated with pneumonia and influenza among non-Hispanic black and white US adults. Am J Public Health.

[54] Allard R, Leclerc P, Tremblay C, Tannenbaum T-N (2010). Diabetes and the severity of pandemic influenza a (H1N1) infection. Diabetes Care.

[55] Wilking H, Buda S, Lippe E, Altmann D, Krause G, Eckmanns T, Haas W (2010). Mortality of 2009 pandemic influenza A (H1N1) in Germany.

[56] Morgan OW, Bramley A, Fowlkes A, Freedman DS, Taylor TH, Gargiullo P, Belay B, Jain S, Cox C, Kamimoto L (2010). Morbid obesity as a risk factor for hospitalization and death due to 2009 pandemic influenza A (H1N1) disease. PLoS One.

[57] Stegenga ME, van der Crabben SN, Blümer RM, Levi M, Meijers JC, Serlie MJ, Tanck MW, Sauerwein HP, van der Poll T (2008). Hyperglycemia enhances coagulation and reduces neutrophil degranulation, whereas hyperinsulinemia inhibits fibrinolysis during human endotoxemia. Blood.

[58] Ilyas R, Wallis R, Soilleux EJ, Townsend P, Zehnder D, Tan BK, Sim RB, Lehnert H, Randeva HS, Mitchell DA (2011). High glucose disrupts oligosaccharide recognition function via competitive inhibition: a potential mechanism for immune dysregulation in diabetes mellitus. Immunobiology.

[59] Alexiewicz JM, Kumar D, Smogorzewski M, Klin M, Massry SG (1995). Polymorphonuclear leukocytes in non-insulin-dependent diabetes mellitus: abnormalities in metabolism and function. Ann Intern Med.

[60] Reading PC, Allison J, Crouch EC, Anders EM (1998). Increased susceptibility of diabetic mice to influenza virus infection: compromise of collectin-mediated host defense of the lung by glucose?. J Virol.

[61] Klemperer H (1961). Glucose breakdown in chick embryo cells infected with influenza virus. Virology.

[62] Thai M, Graham NA, Braas D, Nehil M, Komisopoulou E, Kurdistani SK, McCormick F, Graeber TG, Christofk HR (2014). Adenovirus E4ORF1-induced MYC activation promotes host cell anabolic glucose metabolism and virus replication. Cell Metab.

[63] Janke R, Genzel Y, Wetzel M, Reichl U (2011). Effect of influenza virus infection on key metabolic enzyme activities in MDCK cells. BMC proceedings BioMed Central.

[64] Thomas D, Cherest H, Surdin-Kerjan Y (1991). Identification of the structural gene for glucose-6-phosphate dehydrogenase in yeast. Inactivation leads to a nutritional requirement for organic sulfur. EMBO J.

[65] Hecker PA, Leopold JA, Gupte SA, Recchia FA, Stanley WC (2013). Impact of glucose-6-phosphate dehydrogenase deficiency on the pathophysiology of cardiovascular disease. Am J Phys Heart Circ Phys.

[66] Ho H-Y, Cheng M-L, Weng S-F, Chang L, Yeh T-T, Shih S-R, Chiu DT-Y (2008). Glucose-6-phosphate dehydrogenase deficiency enhances enterovirus 71 infection. J Gen Virol.

[67] Wu Y-H, Tseng C-P, Cheng M-L, Ho H-Y, Shih S-R, Chiu DT-Y (2008). Glucose-6-phosphate dehydrogenase deficiency enhances human coronavirus 229E infection. J Infect Dis.

[68] Friel H, Lederman H (2006). A nutritional supplement formula for influenza a (H5N1) infection in humans. Med Hypotheses.

[69] Kuss-Duerkop Sharon K., Wang Juan, Mena Ignacio, White Kris, Metreveli Giorgi, Sakthivel Ramanavelan, Mata Miguel A., Muñoz-Moreno Raquel, Chen Xiang, Krammer Florian, Diamond Michael S., Chen Zhijian J., García-Sastre Adolfo, Fontoura Beatriz M. A. (2017). Influenza virus differentially activates mTORC1 and mTORC2 signaling to maximize late stage replication. PLOS Pathogens.

[70] Wall M, Poortinga G, Hannan KM, Pearson RB, Hannan RD, McArthur GA (2008). Translational control of c-MYC by rapamycin promotes terminal myeloid differentiation. Blood.

[71] Masui K, Tanaka K, Akhavan D, Babic I, Gini B, Matsutani T, Iwanami A, Liu F, Villa GR, Gu Y (2013). mTOR complex 2 controls glycolytic metabolism in glioblastoma through FoxO acetylation and upregulation of c-Myc. Cell Metab.

[72] Biggs WH, Meisenhelder J, Hunter T, Cavenee WK, Arden KC (1999). Protein kinase B/Akt-mediated phosphorylation promotes nuclear exclusion of the winged helix transcription factor FKHR1. Proc Natl Acad Sci U S A.

[73] Dang CV (2012). Links between metabolism and cancer. Genes Dev.

[74] Dang CV (2012). MYC on the path to cancer. Cell.

[75] Ferber EC, Peck B, Delpuech O, Bell GP, East P, Schulze A (2012). FOXO3a regulates reactive oxygen metabolism by inhibiting mitochondrial gene expression. Cell Death Differ.

[76] Peck B, Ferber EC, Schulze A (2013). Antagonism between FOXO and MYC regulates cellular powerhouse. Front Oncol.

[77] Gordan JD, Thompson CB, Simon MC (2007). HIF and c-Myc: sibling rivals for control of cancer cell metabolism and proliferation. Cancer Cell.

[78] Dang CV, Le A, Gao P (2009). MYC-induced cancer cell energy metabolism and therapeutic opportunities. Clin Cancer Res.

[79] Düvel K, Yecies JL, Menon S, Raman P, Lipovsky AI, Souza AL, Triantafellow E, Ma Q, Gorski R, Cleaver S (2010). Activation of a metabolic gene regulatory network downstream of mTOR complex 1. Mol Cell.

[80] Sun Q, Chen X, Ma J, Peng H, Wang F, Zha X, Wang Y, Jing Y, Yang H, Chen R (2011). Mammalian target of rapamycin up-regulation of pyruvate kinase isoenzyme type M2 is critical for aerobic glycolysis and tumor growth. Proc Natl Acad Sci.

[81] Semenza GL (2010). Defining the role of hypoxia-inducible factor 1 in cancer biology and therapeutics. Oncogene.

[82] Guccini I, Serio D, Condo I, Rufini A, Tomassini B, Mangiola A, Maira G, Anile C, Fina D, Pallone F (2011). Frataxin participates to the hypoxia-induced response in tumors. Cell Death Dis.

[83] Barthel A, Okino ST, Liao J, Nakatani K, Li J, Whitlock JP, Roth RA (1999). Regulation of GLUT1 gene transcription by the serine/threonine kinase Akt1. J Biol Chem.

[84] Rathmell JC, Fox CJ, Plas DR, Hammerman PS, Cinalli RM, Thompson CB (2003). Akt-directed glucose metabolism can prevent Bax conformation change and promote growth factor-independent survival. Mol Cell Biol.

[85] Porstmann T, Santos CR, Griffiths B, Cully M, Wu M, Leevers S, Griffiths JR, Chung Y-L, Schulze A (2008). SREBP activity is regulated by mTORC1 and contributes to Akt-dependent cell growth. Cell Metab.

[86] Sundqvist A, Bengoechea-Alonso MT, Ye X, Lukiyanchuk V, Jin J, Harper JW, Ericsson J (2005). Control of lipid metabolism by phosphorylation-dependent degradation of the SREBP family of transcription factors by SCFFbw7. Cell Metab.

[87] Lin S, Liu N, Yang Z, Song W, Wang P, Chen H, Lucio M, Schmitt-Kopplin P, Chen G, Cai Z (2010). GC/MS-based metabolomics reveals fatty acid biosynthesis and cholesterol metabolism in cell lines infected with influenza a virus. Talanta.

[88] Kliewer SA, Sundseth SS, Jones SA, Brown PJ, Wisely GB, Koble CS, Devchand P, Wahli W, Willson TM, Lenhard JM (1997). Fatty acids and eicosanoids regulate gene expression through direct interactions with peroxisome proliferator-activated receptors α and γ. Proc Natl Acad Sci.

[89] Yu K, Bayona W, Kallen CB, Harding HP, Ravera CP, McMahon G, Brown M, Lazar MA (1995). Differential activation of peroxisome proliferator-activated receptors by eicosanoids. J Biol Chem.

[90] Marion-Letellier R, Savoye G, Ghosh S (2016). Fatty acids, eicosanoids and PPAR gamma. Eur J Pharmacol.

[91] Michalik L, Auwerx J, Berger JP, Chatterjee VK, Glass CK, Gonzalez FJ, Grimaldi PA, Kadowaki T, Lazar MA, O'Rahilly S (2006). International Union of Pharmacology. LXI. Peroxisome proliferator-activated receptors. Pharmacol Rev.

[92] Dunning KR, Anastasi MR, Zhang VJ, Russell DL, Robker RL (2014). Regulation of fatty acid oxidation in mouse cumulus-oocyte complexes during maturation and modulation by PPAR agonists. PLoS One.

[93] Zhang S, Hulver MW, McMillan RP, Cline MA, Gilbert ER (2014). The pivotal role of pyruvate dehydrogenase kinases in metabolic flexibility. Nutr Metab (Lond).

[94] Yamane K, Indalao IL, Chida J, Yamamoto Y, Hanawa M, Kido H (2014). Diisopropylamine dichloroacetate, a novel pyruvate dehydrogenase kinase 4 inhibitor, as a potential therapeutic agent for metabolic disorders and multiorgan failure in severe influenza. PLoS One.

[95] Milner JJ, Rebeles J, Dhungana S, Stewart DA, Sumner SC, Meyers MH, Mancuso P, Beck MA (2015). Obesity increases mortality and modulates the lung Metabolome during pandemic H1N1 influenza virus infection in mice. J Immunol.

[96] Tisoncik-Go J, Gasper DJ, Kyle JE, Eisfeld AJ, Selinger C, Hatta M, Morrison J, Korth MJ, Zink EM, Kim YM (2016). Integrated Omics analysis of pathogenic host responses during pandemic H1N1 influenza virus infection: the crucial role of lipid metabolism. Cell Host Microbe.

[97] Tarasenko TN, Singh LN, Chatterji-Len M, Zerfas PM, Cusmano-Ozog K, McGuire PJ (1852). Kupffer cells modulate hepatic fatty acid oxidation during infection with PR8 influenza. Biochim Biophys Acta.

[98] Cui L, Fang J, Ooi EE, Lee YH (2017). Serial Metabolome changes in a prospective cohort of subjects with influenza viral infection and comparison with dengue fever. J Proteome Res.

[99] Numata M, Kandasamy P, Nagashima Y, Posey J, Hartshorn K, Woodland D, Voelker DR (2012). Phosphatidylglycerol suppresses influenza a virus infection. Am J Respir Cell Mol Biol.

[100] Cui L, Zheng D, Lee YH, Chan TK, Kumar Y, Ho WE, Chen JZ, Tannenbaum SR, Ong CN (2016). Metabolomics investigation reveals metabolite mediators associated with acute lung injury and repair in a murine model of influenza pneumonia. Sci Rep.

[101] Tanner LB, Chng C, Guan XL, Lei Z, Rozen SG, Wenk MR (2014). Lipidomics identifies a requirement for peroxisomal function during influenza virus replication. J Lipid Res.

[102] Espenshade Peter J., Hughes Adam L. (2007). Regulation of Sterol Synthesis in Eukaryotes. Annual Review of Genetics.

[103] Krycer JR, Sharpe LJ, Luu W, Brown AJ (2010). The Akt-SREBP nexus: cell signaling meets lipid metabolism. Trends Endocrinol Metab.

[104] Yang YA, Han WF, Morin PJ, Chrest FJ, Pizer ES (2002). Activation of fatty acid synthesis during neoplastic transformation: role of mitogen-activated protein kinase and phosphatidylinositol 3-kinase. Exp Cell Res.

[105] Yuan S, Chu H, Chan JF-W, Ye Z-W, Wen L, Yan B, Lai P-M, Tee K-M, Huang J, Chen D (2019). SREBP-dependent lipidomic reprogramming as a broad-spectrum antiviral target. Nat Commun.

[106] Bradley-Stewart A, Jolly L, Adamson W, Gunson R, Frew-Gillespie C, Templeton K, Aitken C, Carman W, Cameron S, McSharry C (2013). Cytokine responses in patients with mild or severe influenza a (H1N1) pdm09. J Clin Virol.

[107] Beylot M, Vidal H, Mithieux G, Odeon M, Martin C (1992). Inhibition of hepatic ketogenesis by tumor necrosis factor-alpha in rats. Am J Physiol Endocrinol Metab.

[108] Pailla K, Lim SK, De Bandt JP, Aussel C, Giboudeau J, Troupel S, Cynober L, Blonde-Cynober F (1998). TNF-α and IL-6 synergistically inhibit Ketogenesis from fatty acids and α-Ketoisocaproate in isolated rat hepatocytes. J Parenter Enter Nutr.

[109] Kitade H, Kanemaki T, Sakitani K, Inoue K, Matsui Y, Kamiya T, Nakagawa M, Hiramatsu Y, Kamiyama Y, Ito S, Okumura T (1996). Regulation of energy metabolism by interleukin-1beta, but not by interleukin-6, is mediated by nitric oxide in primary cultured rat hepatocytes. Biochim Biophys Acta.

[110] Yao M, Yao D, Yamaguchi M, Chida J, Yao D, Kido H (2011). Bezafibrate upregulates carnitine palmitoyltransferase II expression and promotes mitochondrial energy crisis dissipation in fibroblasts of patients with influenza-associated encephalopathy. Mol Genet Metab.

[111] Wise DR, DeBerardinis RJ, Mancuso A, Sayed N, Zhang XY, Pfeiffer HK, Nissim I, Daikhin E, Yudkoff M, McMahon SB, Thompson CB (2008). Myc regulates a transcriptional program that stimulates mitochondrial glutaminolysis and leads to glutamine addiction. Proc Natl Acad Sci U S A.

[112] Le Floc'h N, Deblanc C, Cariolet R, Gautier-Bouchardon AV, Merlot E, Simon G (2014). Effect of feed restriction on performance and postprandial nutrient metabolism in pigs co-infected with mycoplasma hyopneumoniae and swine influenza virus. PLoS One.

[113] Laplante M, Sabatini DM (2009). mTOR signaling at a glance. J Cell Sci.

[114] Samuel CE (2001). Influenza virus drug resistance: a time-sampled population genetics perspective. Clin Microbiol Rev.

[115] Fensterl V, Sen GC (2009). Interferons and viral infections. Biofactors.

[116] Burke JD, Platanias LC, Fish EN (2014). Beta interferon regulation of glucose metabolism is PI3K/Akt dependent and important for antiviral activity against Coxsackievirus B3. J Virol.

[117] Wu D, Sanin DE, Everts B, Chen Q, Qiu J, Buck MD, Patterson A, Smith AM, Chang CH, Liu Z (2016). Type 1 Interferons induce changes in Core metabolism that are critical for immune function. Immunity.

[118] Bajwa G, DeBerardinis RJ, Shao B, Hall B, Farrar JD, Gill MA (2016). Cutting edge: critical role of glycolysis in human Plasmacytoid dendritic cell antiviral responses. J Immunol.

[119] Wang F, Zhang S, Jeon R, Vuckovic I, Jiang X, Lerman A, Folmes CD, Dzeja PD, Herrmann J (2018). Interferon gamma induces reversible metabolic reprogramming of M1 macrophages to sustain cell viability and pro-inflammatory activity. EBioMedicine.

[120] Infantino V, Iacobazzi V, Palmieri F, Menga A (2013). ATP-citrate lyase is essential for macrophage inflammatory response. Biochem Biophys Res Commun.

[121] Blanc M, Hsieh WY, Robertson KA, Watterson S, Shui G, Lacaze P, Khondoker M, Dickinson P, Sing G, Rodriguez-Martin S (2011). Host defense against viral infection involves interferon mediated down-regulation of sterol biosynthesis. PLoS Biol.

[122] York AG, Williams KJ, Argus JP, Zhou QD, Brar G, Vergnes L, Gray EE, Zhen A, Wu NC, Yamada DH (2015). Limiting cholesterol biosynthetic flux spontaneously engages type I IFN signaling. Cell.

[123] Brown MS, Goldstein JL (1997). The SREBP pathway: regulation of cholesterol metabolism by proteolysis of a membrane-bound transcription factor. Cell.

[124] Pizzolla A, Smith JM, Brooks AG, Reading PC (2017). Pattern recognition receptor immunomodulation of innate immunity as a strategy to limit the impact of influenza virus. J Leukoc Biol.

[125] Morrison J, Josset L, Tchitchek N, Chang J, Belser JA, Swayne DE, Pantin-Jackwood MJ, Tumpey TM, Katze MG (2014). H7N9 and other pathogenic avian influenza viruses elicit a three-pronged transcriptomic signature that is reminiscent of 1918 influenza virus and is associated with lethal outcome in mice. J Virol.

[126] Short KR, Kroeze EJV, Fouchier RA, Kuiken T (2014). Pathogenesis of influenza-induced acute respiratory distress syndrome. Lancet Infect Dis.

[127] Beigel J (2005). Writing Committee of the World Health Organization Consultation on human influenza a/H5: avian influenza a (H5N1) infection in humans. New Eng J Med.

[128] Kuiken T, Riteau B, Fouchier R, Rimmelzwaan G (2012). Pathogenesis of influenza virus infections: the good, the bad and the ugly. Current opinion in virology.

[129] Wareing MD, Lyon AB, Lu B, Gerard C, Sarawar SR (2004). Chemokine expression during the development and resolution of a pulmonary leukocyte response to influenza a virus infection in mice. J Leukoc Biol.

[130] Kumar Y, Liang C, Limmon GV, Liang L, Engelward BP, Ooi EE, Chen J, Tannenbaum SR (2014). Molecular analysis of serum and bronchoalveolar lavage in a mouse model of influenza reveals markers of disease severity that can be clinically useful in humans. PLoS One.

[131] Marion T, Elbahesh H, Thomas PG, DeVincenzo JP, Webby R, Schughart K (2016). Respiratory mucosal proteome quantification in human influenza infections. PLoS One.

[132] Rogo LD, Rezaei F, Marashi SM, Yekaninejad MS, Naseri M, Ghavami N, Mokhtari-Azad T (2016). Seasonal influenza a/H3N2 virus infection and IL-1Β, IL-10, IL-17, and IL-28 polymorphisms in Iranian population. J Med Virol.

[133] Mounce BC, Poirier EZ, Passoni G, Simon-Loriere E, Cesaro T, Prot M, Stapleford KA, Moratorio G, Sakuntabhai A, Levraud JP, Vignuzzi M (2016). Interferon-induced Spermidine-Spermine Acetyltransferase and polyamine depletion restrict Zika and Chikungunya viruses. Cell Host Microbe.

[134] Yeung AW, Terentis AC, King NJ, Thomas SR (2015). Role of indoleamine 2,3-dioxygenase in health and disease. Clin Sci (Lond).

[135] Boergeling Y, Ludwig S (2017). Targeting a metabolic pathway to fight the flu. FEBS J.

[136] Schmidt SV, Schultze JL (2014). New insights into IDO biology in bacterial and viral infections. Front Immunol.

[137] Fallarino F, Vacca C, Orabona C, Belladonna ML, Bianchi R, Marshall B, Keskin DB, Mellor AL, Fioretti MC, Grohmann U, Puccetti P (2002). Functional expression of indoleamine 2,3-dioxygenase by murine CD8 alpha(+) dendritic cells. Int Immunol.

[138] van Wissen M, Snoek M, Smids B, Jansen HM, Lutter R (2002). IFN-gamma amplifies IL-6 and IL-8 responses by airway epithelial-like cells via indoleamine 2,3-dioxygenase. J Immunol.

[139] Jewell NA, Cline T, Mertz SE, Smirnov SV, Flano E, Schindler C, Grieves JL, Durbin RK, Kotenko SV, Durbin JE (2010). Lambda interferon is the predominant interferon induced by influenza a virus infection in vivo. J Virol.

[140] Ball HJ, Sanchez-Perez A, Weiser S, Austin CJ, Astelbauer F, Miu J, McQuillan JA, Stocker R, Jermiin LS, Hunt NH (2007). Characterization of an indoleamine 2,3-dioxygenase-like protein found in humans and mice. Gene.

[141] Mellor AL, Munn DH (2004). IDO expression by dendritic cells: tolerance and tryptophan catabolism. Nat Rev Immunol.

[142] Munn DH, Mellor AL (2013). Indoleamine 2,3 dioxygenase and metabolic control of immune responses. Trends Immunol.

[143] Huang L, Li L, Klonowski KD, Tompkins SM, Tripp RA, Mellor AL (2013). Induction and role of indoleamine 2,3 dioxygenase in mouse models of influenza a virus infection. PLoS One.

[144] Bogdan C (2015). Nitric oxide synthase in innate and adaptive immunity: an update. Trends Immunol.

[145] Reiss CS, Komatsu T (1998). Does nitric oxide play a critical role in viral infections?. J Virol.

[146] Uehara EU, Shida Bde S, de Brito CA (2015). Role of nitric oxide in immune responses against viruses: beyond microbicidal activity. Inflamm Res.

[147] Akaike T, Noguchi Y, Ijiri S, Setoguchi K, Suga M, Zheng YM, Dietzschold B, Maeda H (1996). Pathogenesis of influenza virus-induced pneumonia: involvement of both nitric oxide and oxygen radicals. Proc Natl Acad Sci.

[148] Uetani K, Der SD, Zamanian-Daryoush M, de la Motte C, Lieberman BY, Williams BR, Erzurum SC (2000). Central role of double-stranded RNA-activated protein kinase in microbial induction of nitric oxide synthase. J Immunol.

[149] Zaki Mohammad Hasan, Akuta Teruo, Akaike Takaaki (2005). Nitric Oxide-Induced Nitrative Stress Involved in Microbial Pathogenesis. Journal of Pharmacological Sciences.

[150] Asano K, Chee C, Gaston B, Lilly CM, Gerard C, Drazen JM, Stamler JS (1994). Constitutive and inducible nitric oxide synthase gene expression, regulation, and activity in human lung epithelial cells. Proc Natl Acad Sci.

[151] Guo FH, De Raeve HR, Rice TW, Stuehr DJ, Thunnissen F, Erzurum SC (1995). Continuous nitric oxide synthesis by inducible nitric oxide synthase in normal human airway epithelium in vivo. Proc Natl Acad Sci.

[152] Sarawar SR, Doherty PC (1994). Concurrent production of interleukin-2, interleukin-10, and gamma interferon in the regional lymph nodes of mice with influenza pneumonia. J Virol.

[153] Takeda N, O'Dea EL, Doedens A, Kim J-W, Weidemann A, Stockmann C, Asagiri M, Simon MC, Hoffmann A, Johnson RS (2010). Differential activation and antagonistic function of HIF-α isoforms in macrophages are essential for NO homeostasis. Genes Dev.

[154] Perrone LA, Belser JA, Wadford DA, Katz JM, Tumpey TM (2013). Inducible nitric oxide contributes to viral pathogenesis following highly pathogenic influenza virus infection in mice. J Infect Dis.

[155] Darwish I, Miller C, Kain KC, Liles WC (2012). Inhaled nitric oxide therapy fails to improve outcome in experimental severe influenza. Int J Med Sci.

[156] Rimmelzwaan G, Baars M, Fouchier R, Osterhaus A. Inhibition of influenza virus replication by nitric oxide. In: International Congress Series: Elsevier; 2001. p. 551–5.. 10.1128/jvi.73.10.8880-8883.1999 PMC112914 10482647

[157] Tamura D, DeBiasi RL, Okomo-Adhiambo M, Mishin VP, Campbell AP, Loechelt B, Wiedermann BL, Fry AM, Gubareva LV (2015). Emergence of multidrug-resistant influenza a(H1N1)pdm09 virus variants in an Immunocompromised child treated with Oseltamivir and Zanamivir. J Infect Dis.

[158] Drakopoulos A, Tzitzoglaki C, Ma C, Freudenberger K, Hoffmann A, Hu Y, Gauglitz GN, Schmidtke M, Wang J, Kolocouris A (2017). Affinity of rimantadine enantiomers against influenza A/M2 protein revisited. ACS Med Chem Lett.

[159] Foll M, Poh YP, Renzette N, Ferrer-Admetlla A, Bank C, Shim H, Malaspinas AS, Ewing G, Liu P, Wegmann D (2014). Influenza virus drug resistance: a time-sampled population genetics perspective. PLoS Genet.

[160] Takanashi K, Dan K, Kanzaki S, Hasegawa H, Watanabe K, Ogawa K (2017). Hochuekkito, a Japanese herbal medicine, restores metabolic homeostasis between mitochondrial and glycolytic pathways impaired by influenza a virus infection. Pharmacology.

[161] Hui KP, Kuok DI, Kang SS, Li HS, Ng MM, Bui CH, Peiris JS, Chan RW, Chan MC (2015). Modulation of sterol biosynthesis regulates viral replication and cytokine production in influenza a virus infected human alveolar epithelial cells. Antivir Res.

[162] Chen L, Fan J, Li Y, Shi X, Ju D, Yan Q, Yan X, Han L, Zhu H (2014). Modified Jiu Wei Qiang Huo decoction improves dysfunctional metabolomics in influenza a pneumonia-infected mice. Biomed Chromatogr.

[163] Yu Jason S. L., Cui Wei (2016). Proliferation, survival and metabolism: the role of PI3K/AKT/mTOR signalling in pluripotency and cell fate determination. Development.

[164] Murray JL, McDonald NJ, Sheng J, Shaw MW, Hodge TW, Rubin DH, O'Brien WA, Smee DF (2012). Inhibition of influenza a virus replication by antagonism of a PI3K-AKT-mTOR pathway member identified by gene-trap insertional mutagenesis. Antivir Chem Chemother.

[165] Su L, Zhang H, Yan C, Chen A, Meng G, Wei J, Yu D, Ding Y (2016). Superior anti-tumor efficacy of diisopropylamine dichloroacetate compared with dichloroacetate in a subcutaneous transplantation breast tumor model. Oncotarget.

